# Should All Electrochemical Energy Materials Be Isomaterially Heterostructured to Optimize Contra and Co-varying Physicochemical Properties?

**DOI:** 10.3389/fchem.2020.00515

**Published:** 2020-06-19

**Authors:** Raj Ganesh S. Pala

**Affiliations:** Department of Chemical Engineering and the Materials Science Programme, Indian Institute of Technology, Kanpur, India

**Keywords:** isomaterial, heterostructure, photoelectrochemistry (PEC), oxygen evolution electrocatalysis, Li-ion battery cathode

## Abstract

Sustainable energy and chemical/material transformation constrained by limited greenhouse gas generation impose a grand challenge and posit outstanding opportunities to electrochemical material devices. Dramatic advancements in experimental and computational methodologies have captured detailed insights into the working of these material devices at a molecular scale and have brought to light some fundamental constraints that impose bounds on efficiency. We propose that the coupling of molecular events in the material device gives rise to contra-varying or co-varying properties and efficiency improving partial decoupling of such properties can be achieved via introducing engineered heterogeneities. A specific class of engineered heterogeneity is in the form of isomaterial heterostructures comprised of non-native and native polymorphs. The non-native polymorph differs from their native/ground state bulk polymorph in terms of its discrete translational symmetry and we anticipate specific symmetry relationships exist between non-native and native structures that enable the formation of interfaces that enhance efficiency. We present circumstantial evidence and provide speculative mechanisms for such an approach with the hope that a more comprehensive delineation of proposed material design will be undertaken.

## Introduction

An inherent elasticity between prosperity and energy/chemical/material usage behooves upon the scientific community to demonstrate sustainable energy and material transformation technologies. This perspective article will focus on a material design strategy that has implications to the electrochemical approach for sustainable development. A grand vision has been envisaged wherein electrochemical and renewable technologies play a central role in energy conversion, chemical and material production with a limited generation of CO_2_ (Seh et al., 2017; De Luna et al., 2019). Central to this strategy are core technologies like solar energy and electrochemical CO_2_ conversion, energy storage in batteries, electrochemical hydrogen generation and oxygen reduction, electrochemical hydrocarbon, and ammonia synthesis (Seh et al., 2017; De Luna et al., 2019). The realization of such a vision will decisively depend on engineered materials and this article focuses on a material design strategy pertaining to photoelectrochemical oxygen evolution reaction (PEC-OER) (Gratzel, 2001; Vayssieres, 2010; Walter et al., 2011; Van de Krol and Gratzel, 2012; Nellist et al., 2016; Sivula and De Krol, 2016; Hellman and Wang, 2017; Mayer, 2017; Pala, 2017; Aslam et al., 2018; Spitler et al., 2020), electrochemical oxygen evolution reaction (EC-OER) (Jung et al., 2016; Saha et al., 2016; Stevens et al., 2017) and cathodes for Lithium-Ion Batteries (C-LIB) (Grey and Tarascon, 2017; Liu et al., 2019).

Dramatic improvements in computing power/theoretical algorithms and the ability to manipulate/characterize materials at the nanoscale has brought about a synergy in experimental design and theoretical analysis of materials properties (Seh et al., 2017). While advances in machine learning are bound to provide enormous data that will increase the odds of identifying an optimal material (Butler et al., 2018), we will still be limited by natural limitations like the abundance of materials in engineering large-area devices widespread in renewable and electrochemical technologies. Over the last two decades, the basic principles underlying many electrochemical processes like Sabatier relationships for electrocatalysis (Nørskov et al., 2014), a molecular description of voltage profile of a discharging LIB (Urban et al., 2016) have been unfolded via density functional theoretical (DFT) simulations guided by close comparison with experiments. Along with such an understanding, the critical bottlenecks like scaling relationship in catalysis (Nørskov et al., 2014), bounds on capacities/voltages/charging kinetics (Grey and Tarascon, 2017) of batteries has clearly illustrated that pathways to the next-generation materials device lie in exploring and engineering the ever-widening material phase space. Often the enhancing efficiency of material devices relies on optimizing “contra-varying” or “co-varying” properties of materials. By contra-varying (co-varying) properties, we refer to pairs of properties like photopotential and photocurrent, bulk and surface energy, reactant and product adsorption energy wherein when one property increases, the complementary property tends to decrease (increase). Typically, optimal performance requires a balance of such contra-varying or co-varying properties and a systematic modulation of such properties provides a strategy for the conceptual design of electrochemical materials. The trade-off in contra-varying or co-varying properties can itself limit the performance of materials and we argue that isomaterially heterostructured interfaces can provide material topologies to scale beyond such limitations. We provide pointers toward the design, synthesis of such interfaces directed toward three specific applications: PEC-OER, EC-OER, and cathodes for LIB. While this perspective article is built on existing literature, we drift into a speculative tone on some topics in the hope that speculation may lead to productive explorations.

## Contra-Varying Properties in PEC-OER, EC-OER and C-LIB and its Consequences

**Figure 1:**
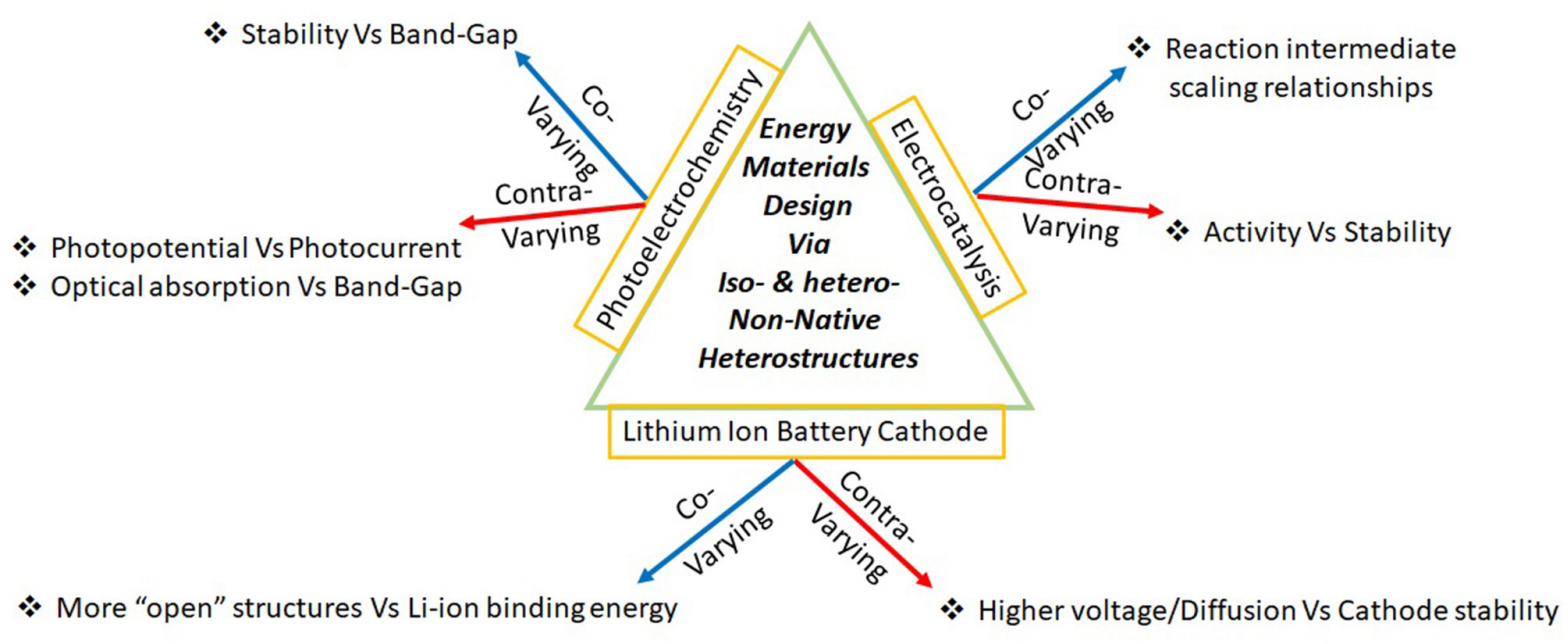
Classifying critical pairs of material properties as covarying or as contravarying facilitates material design via non-native heterostructures.

PEC-OER is an intricate convolution of three processes: optical absorption, electron-hole separation, and surface electrochemistry. This scientifically rich field with immense engineering consequence came to limelight close to five decades ago with the work of Fujishima and Honda (1972), who demonstrated water splitting via TiO_2_ photoanodes. Among the three processes considered in this article, PEC-OER has the greatest number of contra-varying properties (Figure 1) and it is not surprising that this area continues to elude commercialization even after intense explorations. The central challenge of PEC-OER is primarily 3-fold: (1) Poor (good) stability in aqueous solution of materials that show high (low) photoelectrochemical activity (2) High (low) photocurrents are associated with low (high) photopotentials (3) Higher (lower) optical absorption resulting in lower (higher) electron-hole separation. These challenges are intricately connected to the coupled physicochemical processes underlying PEC-OER.

A challenge faced by many electrochemical materials is the issue of stability in aqueous solution and this rationalizes the lesser stability (~5 years, for stationary lead-acid batteries, which are by far the most widely used electrochemical device) of such devices as opposed to other solid-state devices like solar-cells and microprocessors, which have stability ~20 years. The issues of stability are severe in PEC-OER. Typically, most stable materials (like TiO_2_ or ZnO) have large bandgap and hence have lesser optical absorption cross-section and materials like CdS that have greater optical absorption cross-section due to lesser bandgap suffer from photocorrosion. Improving the stability of the photoelectrodes via conformal coatings with optimal transparency and catalytic properties (Moreno-Hernandez et al., 2020) via a coating techniques that are scalable and inexpensive should be explored. The second contra-varying property of photopotential and photocurrent is also due to the issues of bandgap. The deeper (higher) the energy level of the photo-generated hole (electron) in the valence (conduction) band, greater is the driving photooxidation (photoreduction) (Gratzel, 2001; Valdes et al., 2008; Mayer, 2017). The difference between these hole and electron levels provides a measure of photopotential (Gratzel, 2001; Mayer, 2017; Moreno-Hernandez et al., 2020). Typically, materials that generate greater photopotential have higher band-gap and hence absorb lesser photons, leading to lesser photocurrents. Due to these factors, while greater photopotentials provides greater driving force, such materials are inherently poor absorbers. Critical improvements in the measurement of photophotentials via “dual-working electrode” has brought to light the importance of “buried junctions” (Nellist et al., 2016) which will help in more optimal design of the semiconductor/catalyst, catalyst/electrolyte interfaces. Also, the importance of the introduction of the “buried junctions” in limiting the photopotential loss has been demonstrated conclusively recently (Young et al., 2017) and it will be important to extend such efforts via less expensive device fabrication techniques. Much effort in PEC-OER has gone into improving optical absorption of wide bandgap semiconductors (like TiO_2_) and the third contra varying property has implications on this front. Many approaches toward improving optical absorption cross-section like incorporating substitutional dopants, surface grafted-dyes, plasmonic nanoparticles (Vayssieres, 2010; Walter et al., 2010; Behara et al., 2016a; Upadhyay et al., 2016a,b; Pala, 2017; Aslam et al., 2018), hydrogen treatment to make “black-TiO_2_” (Chen et al., 2011; Behara et al., 2014, 2016a,b) increase electronic conductivity and hence, in effect decrease the driving force for electron-hole separation (Behara et al., 2016a). Considering that half-a-century of research into solar water splitting has not yielded a commercial technology, it is worthwhile to consider if the rich science unraveled can be gainfully utilized toward valorising alternate oxidation reactions (Lhermitte and Sivula, 2019) and technologies like semiconductor photoelectrochemical etching (Horikiri et al., 2018).

The main contra-varying property in EC-OER is that the more (less) active OER electrocatalyst are less (more) stable which is due to molecular reasons behind instability involving over-oxidation of cations leading to dissolution into the solutions (Saha et al., 2016). In general, the activity of EC-OER involves the convolution of many properties like atomic oxidation state and electronic conductivity (Gupta et al., 2019), which may also show contra-variation. However, the main challenge in EC-OER is because of the scaling-relationships between different reaction intermediate energies along the multi-electron transfer reaction coordinate (Nørskov et al., 2014), which is due to the covariation of energies of reaction intermediates in a given surface. For a given surface, the energies of different reaction intermediates and transition states in a multi-electron transfer reaction are related to each and hence, it is not easy to dramatically change the activation energies by modifying the surface. This covariation of energies of different reaction intermediates provides a basis for establishing the activity “volcano” as a function of a particular descriptor like adsorption energy of hydroxyl radical over an oxide surface. If such scaling relationships or covariation of energies are broken, then it is anticipated that electrocatalysts will have a greater impact on modifying the activation barriers and hence, the overpotential for OER. Considering the importance of scaling in establishing a rational paradigm for catalyst design, many approaches are being undertaken to break the scaling relationship (Doyle et al., 2015; Vojvodic and Norskov, 2015; Busch et al., 2016; Govindarajan et al., 2019; Perez-Ramirez and Lopez, 2019; Wan et al., 2019; Ting et al., 2020). While the above arguments for the design of electrocatalysts is based on the relative energy levels of the reaction intermediate, the importance of microkinetic analysis has been pointed out recently (Ooka and Nakamura, 2019; Exner, 2020). The important consequence of the microkinetic analysis is to establish that the optimal electrocatalyst achieves thermoneutrality/highest activity volcano peak not at zero overpotential, but at finite operational/onset overpotential (Ooka and Nakamura, 2019; Exner, 2020).

The central contra varying criteria in C-LIB are due to the structural features behind higher voltages/fast charge/discharge characteristics and stability of the electrode (Gupta et al., 2018, 2020). Typically, higher voltages will result from the more negative free energy of intercalation when Li-ion binds to the framework of the cathode. The more open the framework, the more negative the free energy of intercalation, the higher the voltage, facile is the Li-ion diffusion. However, the inherent stability of a more open framework might less (Gupta et al., 2018, 2020). While most of the contemporary commercial Lithium ion battery utilize carbon based anode, LiCoO_2_ or LiMn_2_O_4_ or LiFePO_4_ as cathode (Blomgren, 2017), liquid electrolytes (Blomgren, 2017) and a naturally formed solid-electrolyte-interface (SEI) (Peled and Menkin, 2017), intense research is being directed at other battery chemistries, electrolytes and engineered SEI(Nayak et al., 2018; Wang et al., 2018; Famprikis et al., 2019; Liu et al., 2019; Fu et al., 2020; Manthiram, 2020; Wu et al., 2020) and material interfaces play an critical role in energetics and kinetics of LIB.

## Isomaterially Heterostructures Involving Non-Native Structures

Having obtained certain level of clarity in the underlying bottlenecks on the optimization of electrochemical processes, a variety of approaches are being undertaken to go beyond the constraints. Specifically, our group has been exploring the design, synthesis, characterization and demonstration of “non-native” structures Pandey and Pala, 2012, 2013a,b; Behara et al., 2016b; Upadhyay et al., 2016b; Pala, 2017; Bhattacharya et al., 2018; Gupta et al., 2018, 2019, 2020; Rashmi et al., 2019; Saha et al., 2020). The many non-native structures and most stable “native” bulk structures differ in their discrete translational symmetry and energetic stability (Pandey and Pala, 2012). For e.g., for large crystals of TiO_2_, the most stable bulk phase has Rutile structure and the Anatase structure forms the non-native structure. As particle energies are dependent on the nanoparticle and crystallite size, a classification based solely on metastability is ambiguous and so we have adopted discrete translational symmetry as the central characterizing feature to label structures. Also, the traditional name of different polymorphic structures (like α/β/γ or Ramsdellite) does not readily give information about the thermodynamic stability and hence, we have adopted the notation like NN2, NN1, and N, where the energetic stability is in the order NN2 < NN1 < N (Gupta et al., 2018, 2019, 2020). Typically, material devices made of higher non-native structure is expected to be less stable than those made of lower non-native or native (Gupta et al., 2018, 2019, 2020). We anticipate that book-keeping discrete translational symmetry will also allow for a systematic approach toward the design of interfaces in terms of group-subgroup symmetry elements within the space-group theory (Rashmi et al., 2019). A control on the discrete translational symmetry and the chemical coordination allows for the modulation of properties like bandgap, band-alignment, interfacial coordination structure, bulk structure relevant to ion-intercalation, and diffusion (Gupta et al., 2018, 2019, 2020; Rashmi et al., 2019).

The physical principles underlying the stabilization of non-native structures and some of the relevant material synthetic techniques have been delineated in the literature and will not be unfolded in this article (Upadhyay et al., 2016b; Bhattacharya et al., 2018; Gupta et al., 2018, 2019, 2020; Saha et al., 2020). Central to the stabilization of non-native structure is the relative differences in surface and bulk energy w.r.t the native structure. The surface energy can be modulated via ligands of various kinds and the bulk energy can be modulated via variables like pressure, temperature and chemical strain. Different synthetic approaches like the template, ligand, precursor, dopant, temperature, pressure-assisted stabilization, electrochemical deposition, and exfoliation have been utilized for assembling non-native structures. Once a phase pure non-native crystalline structure has been formed, thermally arrested phase transitions can be utilized to modulate the extent of non-native/native structure in a biphasic material and this also provides modulation of the interface between the non-native and native structures (Gupta et al., 2018, 2019, 2020). In addition to thermal modulation, approaches via “click-chemistry” can also be utilized to assemble interfaces (Upadhyay et al., 2013, 2016b; Behara et al., 2016b), especially if the electron and hole transfer properties of different kinds of click-bonds/clicked-interfaces are better understood. If hole and electron transfer via a clicked interface is facile, clicked interfaces may offer a broader range of interfaces unconstrained by lattice-mismatch and issues of misfit strain at the interface.

In the last two decades, an essential approach to improving the efficiency of PEC-OER is to utilize type-2 heterostructures (Chakrapani, 2014; Li et al., 2014; Rashmi et al., 2019). Such heterostructures can improve optical absorption because of having two semiconductors with distinct bandgaps, will promote electron-hole separation due to staggering of the valence band edge (VBE) and conduction band edge (CBE) (Chakrapani, 2014; Li et al., 2014; Rashmi et al., 2019). Having two distinct semiconductors can also allow for different cathodic and anodic surface sites on either side of the heterostructures. While there has been much emphasis on type-2 heterostructures based on the position of bulk band-edges, a recent study we undertook demonstrates that broken interfacial bonds may vitiate the ability of the heterostructure to separate electron-hole pair (Rashmi et al., 2019). Our study suggests that once the discrete translational symmetry and constraints of complementary chemical bonding are taken into account, the number of possible type-2 semiconducting interfaces is dramatically reduced from what is apparent from the position of CBE and VBE in bulk (Rashmi et al., 2019). We believe that the design of type-2 heterostructures can be made more efficient by adopting the framework of isomaterial non-native/native heterostructures. In such systems, a coherent interface (avoiding broken interfacial bonds) with less strain can be achieved along certain Miller indices. The avoidance of broken interfacial bonds not only gives rise to better electron-hole separation, but it should also result in less interfacial electronic defect generated conductivity and hence, better sustain the driving force obtained from staggered CBE/VBE. Being isomaterial, we anticipate that trends in CBE and VBE can also be systematized to ascertain which semiconductor pair can form a type-2 heterostructure. Besides, trends in CBE/VBE can also be utilized for anticipating stability in aqueous solution. Some of these ideas have been implemented in work from our group (Upadhyay et al., 2013, 2016b; Behara et al., 2016b; Pala, 2017; Rashmi et al., 2019). While we have focused mainly on transition metal oxide materials for PEC, much of the non-native crystal engineering and design principles can be readily generalized to other compounds like chalcogenides and fluorites, which we have explored for other optoelectronic applications (Pala et al., 2009; Pandey and Pala, 2013a; Rastogi et al., 2017; Bhattacharya et al., 2018; Saha et al., 2018). It will be useful to investigate heuristics for coherent interfaces between an inner (i.e. interfacing with the current collector) non-native structures and outer (i.e., interfacing with the electrolyte) stable inorganic crystal, wherein the outer crystal acts as conformal coating by stabilizing the non-native structure not only because it acts as a barrier against corrosion but also because it has a favorable interfacial energy with the non-native structure. Also, a semiconductor-cocatalyst configuration wherein the cocatalyst is stabilized in the non-native form can also be explored.

We envisage two advantages of using isomaterial non-native/native heterostructures for EC-OER. First, it is seen that activity for EC-OER depends not only on the surface electronic structure but also on the bulk electronic structure as the latter determines electronic conductivity. Isomaterially heterostructured non-native/native systems can enable the design of such a biphasic system wherein one structure has optimized surface electronic structure for electrocatalysis and the other structure provides better conductivity. Such a biphasic crystalline assembly need not necessarily be of core-shell architecture but may have interleaving non-native and native crystalline phases, which may allow for breaking of constrains of scaling relationships between different intermediate steps. We speculate that in a reaction proceeding via a Langmuir-Hinshelwood mechanism, reaction intermediates can move between the non-native and native phases in such a manner to break the scaling relationship. Being isomaterially heterostructured with a coherent interface, there is a higher chance for rapid diffusion between the non-native and native phases as the potential energy surface will be less corrugated without the presence of broken bonds at the interface between the non-native and native crystallites. Of course, a systematic conceptual design of material backed by comprehensive experiments is required to realize these speculative suggestions, part of which has been explored in our group with different non-native structures of MnO_2_ (Gupta et al., 2019). Such principles are not only restricted to heterostructured metal oxide interfaces, but might be of relevance to polycrystalline metallic grain boundaries too (Mariano et al., 2017), wherein grain-boundaries might take a non-native structure, especially since stress/strain field is expected to play a role in metal polymorphism (Brog et al., 2013).

Lastly, isomaterial heterostructured non-native and native structures can also be beneficial in C-LIB (Gupta et al., 2018, 2020). Typically, the non-native structures are more open structures and hence, will not only have better Li-ion diffusion kinetics, but also have higher discharge potential as the intercalation binding energy will be more negative (Gupta et al., 2018, 2020). However, the lesser stability of non-native structures will result in considerable voltage fading and this can be diminished by scaffolding the non-native structure with the more stable native structure (Gupta et al., 2018, 2020). In this architecture, the ideal C-LIB is obtained as a trade-off between higher-voltage/faster kinetics and stability by interlacing non-native and native structures. We have implemented such a strategy in C-LIB with MnO_2_ and we found that in addition to a higher voltage, faster kinetics, there was an additional benefit of broader voltage-plateau because the interlacing of non-native structures with the native structure created additional intercalation sites with higher voltages (Gupta et al., 2018, 2020). In general, non-native structures can also be expected to play a role in LIB cathode properties that are crucially controlled by facet and interfacial engineering of the transitional metal oxides (Yao et al., 2019; Zou et al., 2020).

## Conclusion

It is said that the famous Chinese quote “May you live in interesting times” is partly a curse and going by estimates of the enormity of climate crisis (Palmer and Stevens, 2019) and mass extinction rates (Alroy, 2015), the statement is very true of the times we live. While advances in experimental and computational methodology have brought in an exquisitely detailed understanding of many working principles of electrochemical material devices, finer optimization of materials, and chemical processes is required for the scientific response to contemporary challenges. An approach pioneered in the optimization of chemical engineering processes is via “unit operations” (McCabe et al., 2005) wherein an integrated chemical process like reactive-distillation is deconvoluted into reactions in a reactor and separations in a distillation column. Deconvoluting macroscopic chemical processes allows for the operation of the individual unit operations at different conditions/pressures/temperatures to better facilitate optimization of individual unit operations. While process integration has its advantages (Stankiewicz and Moulijn, 2000), it not only introduces challenges in process control but also imposes same process condition for implementing different aspects of overall chemical/material transformation.

At the material level too, optimization is better achieved when the critical physicochemical events can at least be partially deconvoluted to enable decoupled modulation. However, at the material level, unlike the process level unit operations, it is harder to disassemble processes at the molecular level to permit optimization of individual elements. In some instances, the operational procedures for deconvolution is straightforward. E.g., a photoelectrochemical process can be disassembled at a material level by separating photon absorption and electron-hole splitting (via solar cells) from electrochemical processes (via electrolyzer), allowing for better optimization of the two material components separately. Coupling of physicochemical events impose fundamental bounds on efficiencies and may give rise to contra varying (Photopotential vs. Photocurrent, Electrocatalytic Activity vs. Stability, Battery voltage vs. voltage fading) or co-varying relationships (Activation barriers in a multi-electron transfer step) that makes engineering processes at a molecular scale challenging.

We propose that decoupling can be implemented via engineering heterogeneity in the material in the form of isomaterially heterostructured interfaces comprised of non-native and native crystallographic structures. A variety of non-native structures have been synthesized and well-characterized by modulating the surface and bulk energies. Such interfaces will be related via group-subgroup relationship within space-group theory due to which there is a higher chance of having interfaces bound coherently without broken interfacial bonds, which in turn aid in the efficiency of material devices. Much of the important details of such an approach remains to be worked out and corroborated via experiments and we hope that this article will aid in further exploration along this direction.

## Author Contributions

The author confirms being the sole contributor of this work and has approved it for publication.

## Conflict of Interest

The author declares that the research was conducted in the absence of any commercial or financial relationships that could be construed as a potential conflict of interest.

## References

[B1] Alroy J. (2015). Current extinction rates of reptiles and amphibians. Proc. Natl. Acad. Sci. U.S.A. 112, 13003–13008. 10.1073/pnas.1508681112 26438855 PMC4620882

[B2] Aslam U. Rao V. G. Chavez S. Linic S. (2018). Catalytic conversion of solar to chemical energy on plasmonic metal nanostructures. Nat. Catal. 1, 656–665. 10.1038/s41929-018-0138-x

[B3] Behara D. K. Saha S. Babu R. Das M. K. S S. Pala R. G. S. (2014). Design of photoelectrohemical materials via non-native nanostructures and their “click” assembly into photoreactor. SMC Bull 5, 49–58.

[B4] Behara D. K. Sharma G. P. Upadhyay A. P. Gyanprakash M. Pala R. G. S. Sri S. (2016b). Synchronization of charge carrier separation by tailoring the interface of Si-Au-TiO2 heterostructures via click chemistry for PEC water splitting. Chem. Eng. Sci. 154, 150–169. 10.1016/j.ces.2016.06.063

[B5] Behara D. K. Ummireddi A. K. Aragonda V. Gupta P. K. Pala R. G. S. Sivakumara S. (2016a). Coupled optical absorption, charge carrier separation, and surface electrochemistry in surface disordered/hydrogenated TiO2 for enhanced PEC water splitting reaction. Phys. Chem. Chem. Phys. 18, 8364–8377. 10.1039/C5CP04212G 26898750

[B6] Bhattacharya D. Saha S. Shrivastava V. P. Pala R. G. S. Sivakumar S. (2018). Designing coupled quantum dots with ZnS-CdSe hybrid structures for enhancing exciton lifetime. J. Phys. Chem. C 122, 9198–9208. 10.1021/acs.jpcc.8b01210

[B7] Blomgren G. E. (2017). The development and future of lithium Ion batteries. J. Electrochem. Soc. 164, A5019–A5025. 10.1149/2.0251701jes

[B8] Brog J.-P. Chanez C.-L. Crochet A. Fromm K. M. (2013). Polymorphism, what it is and how to identify it: a systematic review. RSC Adv. 3, 16905–16931. 10.1039/c3ra41559g

[B9] Busch M. Halck N. B. Kramm U. I. Siahrostami S. Krtil P. Rossmeisl J. (2016). Beyond the top of the volcano? A unified approach to electrocatalytic oxygen reduction and oxygen evolution. Nano Energy 29, 126–135. 10.1016/j.nanoen.2016.04.011

[B10] Butler K. T. Davies D. W. Cartwright H. Isayev O. Walsh A. (2018). Machine learning for molecular and materials science. Nature 559, 547–555. 10.1038/s41586-018-0337-2 30046072

[B11] Chakrapani V. (2014). “Semiconductor junctions, solid-solid junctions,” in Encyclopedia of Applied Electrochemistry, eds G. Kreysa, K. I. Ota, and R. F. Savinell (New York, NY: Springer), 1882–1893. 10.1007/978-1-4419-6996-5_44

[B12] Chen X. B. Liu L. Yu P. Y. Mao S. S. (2011). Increasing solar absorption for photocatalysis with black hydrogenated titanium dioxide nanocrystals. Science 331, 746–750. 10.1126/science.1200448 21252313

[B13] De Luna P. Hahn C. Higgins D. Jaffer S. A. Jaramillo T. F. Sargent E. H. (2019). What would it take for renewably powered electrosynthesis to displace petrochemical processes? Science 364:eaav3506. 10.1126/science.aav3506 31023896

[B14] Doyle A. D. Montoya J. H. Vojvodic A. (2015). Improving oxygen electrochemistry through nanoscopic confinement. ChemCatChem 7, 738–742. 10.1002/cctc.201402864

[B15] Exner K. S. (2020). Electrolyte engineering as a key strategy towards a sustainable energy scenario? Chemelectrochem 7, 594–595. 10.1002/celc.201902009

[B16] Famprikis T. Canepa P. Dawson J. A. Islam M. S. Masquelier C. (2019). Fundamentals of inorganic solid-state electrolytes for batteries. Nat. Mater. 18, 1278–1291. 10.1038/s41563-019-0431-3 31427742

[B17] Fu C. Venturi V. Kim J. Ahmad Z. Ells A. W. Viswanathan V.. (2020). Universal chemomechanical design rules for solid-ion conductors to prevent dendrite formation in lithium metal batteries. Nat. Mater. 1–9. 10.1038/s41563-020-0655-2 32341510

[B18] Fujishima A. Honda K. (1972). Electrochemical photolysis of water at a semiconductor electrode. Nature 238:37. 10.1038/238037a0 12635268

[B19] Govindarajan N. Koper M. T. M. Meijer E. J. Calle-Vallejo F. (2019). Outlining the scaling-based and scaling-free optimization of electrocatalysts. ACS Catal. 9, 4218–4225. 10.1021/acscatal.9b00532

[B20] Gratzel M. (2001). Photoelectrochemical cells. Nature 414, 338–344. 10.1038/35104607 11713540

[B21] Grey C. P. Tarascon J. M. (2017). Sustainability and in situ monitoring in battery development. Nat. Mater. 16, 45–56. 10.1038/nmat4777 27994251

[B22] Gupta P. K. Bhandari A. Bhattacharya J. Pala R. G. S. (2018). Correlating voltage profile to molecular transformations in ramsdellite MnO2 and its implication for polymorph engineering of lithium Ion battery cathodes. J. Phys. Chem. C 122, 11689–11700. 10.1021/acs.jpcc.8b02708

[B23] Gupta P. K. Bhandari A. Bhattacharya J. Pala R. G. S. (2020). Higher voltage, wider voltage plateau, longer cycle life, and faster kinetics via thermally modulated intergrowth structure of ramsdellite and pyrolusite MnO2 for lithium-ion battery cathodes. J. Power Sour. 450:227619 10.1016/j.jpowsour.2019.227619

[B24] Gupta P. K. Bhandari A. Saha S. Bhattacharya J. Pala R. G. S. (2019). Modulating oxygen evolution reactivity in MnO2 through polymorphic engineering. J. Phys. Chem. C 123, 22345–22357. 10.1021/acs.jpcc.9b05823

[B25] Hellman A. Wang B. (2017). First-principles view on photoelectrochemistry: water-splitting as case study. Inorganics 5:37 10.3390/inorganics5020037

[B26] Horikiri F. Ohta H. Asai N. Narita Y. Yoshida T. Mishima T. (2018). Excellent potential of photo-electrochemical etching for fabricating high-aspect-ratio deep trenches in gallium nitride. Appl. Phys. Expr. 11:091001 10.7567/APEX.11.091001

[B27] Jung S. McCrory C. C. L. Ferrer I. M. Peters J. C. Jaramillo T. F. (2016). Benchmarking nanoparticulate metal oxide electrocatalysts for the alkaline water oxidation reaction. J. Mater. Chem. A 4, 3068–3076. 10.1039/C5TA07586F

[B28] Lhermitte C. R. Sivula K. (2019). Alternative oxidation reactions for solar-driven fuel production. ACS Catal. 9, 2007–2017. 10.1021/acscatal.8b04565

[B29] Li L. Salvador P. A. Rohrer G. S. (2014). Photocatalysts with internal electric fields. Nanoscale 6, 24–42. 10.1039/C3NR03998F 24084897

[B30] Liu J. Bao Z. N. Cui Y. Dufek E. J. Goodenough J. B. Khalifah P. (2019). Pathways for practical high-energy long-cycling lithium metal batteries. Nat. Energy 4, 180–186. 10.1038/s41560-019-0338-x

[B31] Manthiram A. (2020). A reflection on lithium-ion battery cathode chemistry. Nat. Commun. 11, 1–9. 10.1038/s41467-020-15355-0 32214093 PMC7096394

[B32] Mariano R. G. McKelvey K. White H. S. Kanan M. W. (2017). Selective increase in CO_2_ electroreduction activity at grain-boundary surface terminations. Science 358, 1187–1192. 10.1126/science.aao3691 29191908

[B33] Mayer M. T. (2017). Photovoltage at semiconductor-electrolyte junctions. Curr. Opin. Electrochem. 2, 104–110. 10.1016/j.coelec.2017.03.006

[B34] McCabe W. Smith J. Harriott P. (2005). Unit Operations of Chemical Engineering. New York, NY: McGraw-Hill Education.

[B35] Moreno-Hernandez I. A. Yalamanchili S. Fu H. J. Atwater H. A. Brunschwig B. S. Lewis N. S. (2020). Conformal SnOx heterojunction coatings for stabilized photoelectrochemical water oxidation using arrays of silicon microcones. J. Mater. Chem. A 8, 9292–9301. 10.1039/D0TA01144D

[B36] Nayak P. K. Erickson E. M. Schipper F. Penki T. R. Munichandraiah N. Adelhelm P. (2018). Review on challenges and recent advances in the electrochemical performance of high capacity Li- and Mn-rich cathode materials for Li-Ion batteries. Adv. Energy Mater. 8:1702397 10.1002/aenm.201702397

[B37] Nellist M. R. Laskowski F. A. L. Lin F. D. Mills T. J. Boettcher S. W. (2016). Semiconductor-electrocatalyst interfaces: theory, experiment, and applications in photoelectrochemical water splitting. Acc. Chem. Res. 49, 733–740. 10.1021/acs.accounts.6b00001 27035051

[B38] Nørskov J. K. Studt F. Abild-Pedersen F. Bligaard T. (2014). Fundamental Concepts in Heterogeneous Catalysis. Hoboken, NJ: Wiley. 10.1002/9781118892114

[B39] Ooka H. Nakamura R. (2019). Shift of the optimum binding energy at higher rates of catalysis. J. Phys. Chem. Lett. 10, 6706–6713. 10.1021/acs.jpclett.9b01796 31625745

[B40] Pala R. (2017). “Solar hydrogen as a “renewable reductant”: points and counterpoints,” in *CRC Handbook of Thermal Engineering. 2nd Edn* ed R. P. Chhabra (Boca Raton, FL: CRC Press), 1369–1385.

[B41] Pala R. G. S. Tang W. Sushchikh M. M. Park J. N. Forman A. J. Wu G. (2009). CO oxidation by Ti- and Al-doped ZnO: oxygen activation by adsorption on the dopant. J. Catal. 266, 50–58. 10.1016/j.jcat.2009.05.011

[B42] Palmer T. Stevens B. (2019). The scientific challenge of understanding and estimating climate change. Proc. Natl. Acad. Sci. U.S.A. 116, 24390–24395. 10.1073/pnas.1906691116 31792170 PMC6900733

[B43] Pandey M. Pala R. G. S. (2012). Stabilization and growth of non-native nanocrystals at low and atmospheric pressures. J. Chem. Phys. 136:044703. 10.1063/1.3678181 22299910

[B44] Pandey M. Pala R. G. S. (2013a). Stabilization of rocksalt CdSe at atmospheric pressures via pseudomorphic growth. J. Phys. Chem. C 117, 7643–7647. 10.1021/jp400295n

[B45] Pandey M. Pala R. G. S. (2013b). Hydroxylation induced stabilization of near-surface rocksalt nanostructure on wurtzite ZnO structure. J. Chem. Phys. 138:224701. 10.1063/1.4809526 23781809

[B46] Peled E. Menkin S. (2017). Review-SEI: past, present and future. J. Electrochem. Soc. 164, A1703–A1719. 10.1149/2.1441707jes

[B47] Perez-Ramirez J. Lopez N. (2019). Strategies to break linear scaling relationships. Nat. Catal. 2, 971–976. 10.1038/s41929-019-0376-6

[B48] Rashmi Sivakumar S. Pala R. G. S. (2019). Coherency and lattice misfit strain critically constrains electron-hole separation in isomaterial and heteromaterial type-II heterostructures. J. Phys. Chem. C 123, 28620–28630. 10.1021/acs.jpcc.9b08451

[B49] Rastogi C. K. Sharma S. K. Patel A. Parthasarathy G. Pala R. G. S. Kumar J. (2017). Dopant induced stabilization of metastable zircon-type tetragonal LaVO4. J. Phys.Chem. C 121, 16501–16512. 10.1021/acs.jpcc.7b04508

[B50] Saha S. Gupta P. K. Pala R. (2020). Stabilization of Non-Native Polymorphs for Electrocatalysis and Energy Storage Systems. Hoboken, NJ: WIREs Energy and Environment.

[B51] Saha S. Kishor K. Sivakumar S. Pala R. G. S. (2016). Models and mechanisms of oxygen evolution reaction on electrocatalytic surface. J. Indian Inst. Sci. 96, 325–349.

[B52] Saha S. Pala R. G. S. Siyakumar S. (2018). Catalyzing cubic-to-hexagonal phase transition in NaYF4 via ligand enhanced surface ordering. Cryst. Growth Des. 18, 5080–5088. 10.1021/acs.cgd.8b00535

[B53] Seh Z. W. Kibsgaard J. Dickens C. F. Chorkendorff I. B. Norskov J. K. Jaramillo T. F. (2017). Combining theory and experiment in electrocatalysis: insights into materials design. Science 355:eaad4998. 10.1126/science.aad4998 28082532

[B54] Sivula K. De Krol R. V. (2016). Semiconducting materials for photoelectrochemical energy conversion. Nat. Rev. Mater. 1:15010 10.1038/natrevmats.2015.10

[B55] Spitler M. T. Modestino M. A. Deutsch T. G. Xiang C. X. X. Durrant J. R. Esposito D. V. (2020). Practical challenges in the development of photoelectrochemical solar fuels production. Sust Energy Fuels 4, 985–995. 10.1039/C9SE00869A

[B56] Stankiewicz A. I. Moulijn J. A. (2000). Process intensification: transforming chemical engineering. Chem. Eng. Prog. New York, NY, 96, 22–33.

[B57] Stevens M. B. Enman L. J. Batchellor A. S. Cosby M. R. Vise A. E. Trang C. D. M. (2017). Measurement techniques for the study of thin film heterogeneous water oxidation electrocatalysts. Chem. Mater. 29, 120–140. 10.1021/acs.chemmater.6b02796

[B58] Ting L. R. L. Pique O. Lim S. Y. Tanhaei M. Calle-Vallejo F. Yeo B. S. (2020). Enhancing CO2 electroreduction to ethanol on copper-silver composites by opening an alternative catalytic pathway. ACS Catal. 10, 4059–4069. 10.1021/acscatal.9b05319

[B59] Upadhyay A. P. Behara D. K. Sharma G. P. Bajpai A. Sharac N. Ragan R.. (2013). Generic process for highly stable metallic nanoparticle-semiconductor heterostructures via click chemistry for electro/photocatalytic applications. ACS Appl. Mater. Interfaces 5, 9554–9562. 10.1021/am402398h 24018108

[B60] Upadhyay A. P. Behara D. K. Sharma G. P. Gyanprakash M. Pala R. G. S. Sivakumar S. (2016b). Fabricating appropriate band-edge-staggered heterosemiconductors with optically activated Au nanoparticles via click chemistry for photoelectrochemical water splitting. ACS Sust. Chem. Eng. 4, 4511–4520. 10.1021/acssuschemeng.6b00335

[B61] Upadhyay A. P. Rastogi C. K. Pala R. G. S. Sivakumar S. (2016a). Desorption retarded optically complemented multiple dye-sensitized photoelectrochemical water splitting system with enhanced performance. Int. J. Hydrogen Energy 41, 10727–10736. 10.1016/j.ijhydene.2016.04.200

[B62] Urban A. Seo D. H. Ceder G. (2016). Computational understanding of Li-ion batteries. NPJ Comput. Mater. 2:16002 10.1038/npjcompumats.2016.2

[B63] Valdes A. Qu Z. W. Kroes G. J. Rossmeisl J. Norskov J. K. (2008). Oxidation and photo-oxidation of water on TiO2 surface. J. Phys. Chem. C 112, 9872–9879. 10.1021/jp711929d

[B64] Van de Krol R. Gratzel M. (2012). Photoelectrochemical Hydrogen Production. Boston, MA: Springer 10.1007/978-1-4614-1380-6

[B65] Vayssieres L. (2010). On Solar Hydrogen and Nanotechnology. Singapore: John Wiley & Sons 10.1002/9780470823996

[B66] Vojvodic A. Norskov J. K. (2015). New design paradigm for heterogeneous catalysts. Natl. Sci. Rev. 2, 140–143. 10.1093/nsr/nwv023

[B67] Walter M. G. Warren E. L. McKone J. R. Boettcher S. W. Mi Q. Santori E. A.. (2010). Solar water splitting cells. Chem. Rev. 110:6446. 10.1021/cr1002326 21062097

[B68] Walter M. G. Warren E. L. McKone J. R. Boettcher S. W. Mi Q. X. Santori E. A.. (2011). Solar water splitting cells. Chem. Rev. 110, 6446–6473. 10.1021/cr200102n 21062097

[B69] Wan H. Ostergaard T. M. Arnarson L. Rossmeisl J. (2019). Climbing the 3D volcano for the oxygen reduction reaction using porphyrin motifs. ACS Sust. Chem. Eng. 7, 611–617. 10.1021/acssuschemeng.8b04173

[B70] Wang A. P. Kadam S. Li H. Shi S. Q. Qi Y. (2018). Review on modeling of the anode solid electrolyte interphase (SEI) for lithium-ion batteries. NPJ Comput. Mater. 4, 1–26. 10.1038/s41524-018-0064-0

[B71] Wu F. X. Maier J. Yu Y. (2020). Guidelines and trends for next-generation rechargeable lithium and lithium-ion batteries. Chem. Soc. Rev. 49, 1569–1614. 10.1039/C7CS00863E 32055806

[B72] Yao W. Yuan Y. Tan G. Liu C. Cheng M. Yurkiv V.. (2019). Tuning Li2O2 formation routes by facet engineering of MnO2 cathode catalysts. J. Am. Chem. Soc. 141, 12832–12838. 10.1021/jacs.9b05992 31334638

[B73] Young J. L. Steiner M. A. Doscher H. France R. M. Turner J. A. Deutsch T. G. (2017). Direct solar-to-hydrogen conversion via inverted metamorphic multi-junction semiconductor architectures. Nat. Energy 2:17028 10.1038/nenergy.2017.28

[B74] Zou L. Zhao W. Jia H. Zheng J. Li L. Abraham D. P. (2020). The role of secondary particle structures in surface phase transitions of Ni-Rich cathodes. Chem. Mater. 32, 2884–2892. 10.1021/acs.chemmater.9b04938

